# Long‐term follow‐up with filter paper samples in patients with propionic acidemia

**DOI:** 10.1002/jmd2.12166

**Published:** 2020-09-27

**Authors:** Sinziana Stanescu, Amaya Belanger‐Quintana, Borja Manuel Fernández‐Felix, Celia Pérez‐Cerdá, Begoña Merinero, Pedro Ruiz‐Sala, Francisco Arrieta, Mercedes Martínez‐Pardo

**Affiliations:** ^1^ Unidad de Enfermedades Metabólicas Hospital Universitario Ramón y Cajal, IRYCIS, CIBER‐OBN Madrid Spain; ^2^ Unidad de Bioestadística Clínica Instituto Ramón y Cajal de Investigación Sanitaria, Hospital Universitario Ramón y Cajal Madrid Spain; ^3^ Centro de Diagnóstico de Enfermedades Moleculares, Centro de Biología Molecular Universidad Autónoma de Madrid, CIBERER, IdiPAZ Madrid Spain

**Keywords:** filter paper samples, follow‐up, propionic acidemia

## Abstract

**Background:**

Propionic acidemia (PA) is an inherited disorder caused by deficiency of propionyl CoA carboxylase. Most patients with this disorder are diagnosed during the neonatal period because of severe metabolic acidosis and hyperammonemia. Patients are required to undergo blood and urine analysis at least 3 to 4 times per year, depending on age and metabolic control.

**Methods:**

We designed a prospective study in which we investigated the results from blood and urinary samples collected monthly in filter paper from 10 PA patients followed in a single metabolic reference center from January 2015 to September 2017. The aim of this study was to evaluate the usefulness of filter paper samples in the follow‐up of the PA patients.

**Results:**

During the follow‐up period, 163 dried blood spot (DBS) and 119 urine dried spot samples were analyzed and compared with 160 plasma and 103 liquid urine specimens; 64 specimens of plasma were analyzed for odd‐numbered long‐chain fatty acids (OLCFAs). A total of 40 metabolic crises, 18 of them with hyperammonemia were documented. We observed a strong correlation between the filter paper and the urine/plasma samples for the main PA parameters both in stable metabolic conditions as well as in acute decompensations. Also, there was a strong correlation between OLCFAs measured in plasma and quantification of odd number acylcarnitines in DBS.

**Conclusions:**

We conclude that filter paper blood and urinary samples can be used for the follow‐up of the patients with PA, correctly reflecting their metabolic situation.

**Synopsis**

The filter paper samples can be used for the follow‐up of the patients with propionic acidemia, correctly reflecting their metabolic situation.

## 1 INTRODUCTION

Propionic acidemia (PA; OMIM 606054) is a rare autosomal recessive disease with an estimated prevalence of 1:100 000 to 150 000.^1^ It is caused by a deficiency of propionyl CoA carboxylase (PCC), a mitochondrial enzyme that converts the propionyl CoA to methylmalonyl CoA, which will enter in the citric acid cycle to generate ATP. Propionyl CoA is formed by degradation of several essential amino acids, cholesterol, and odd‐numbered long‐chain fatty acids (OLCFAs). Also, important amounts of propionic acid are produced by the gut bacteria.^1^

**FIGURE 1:**
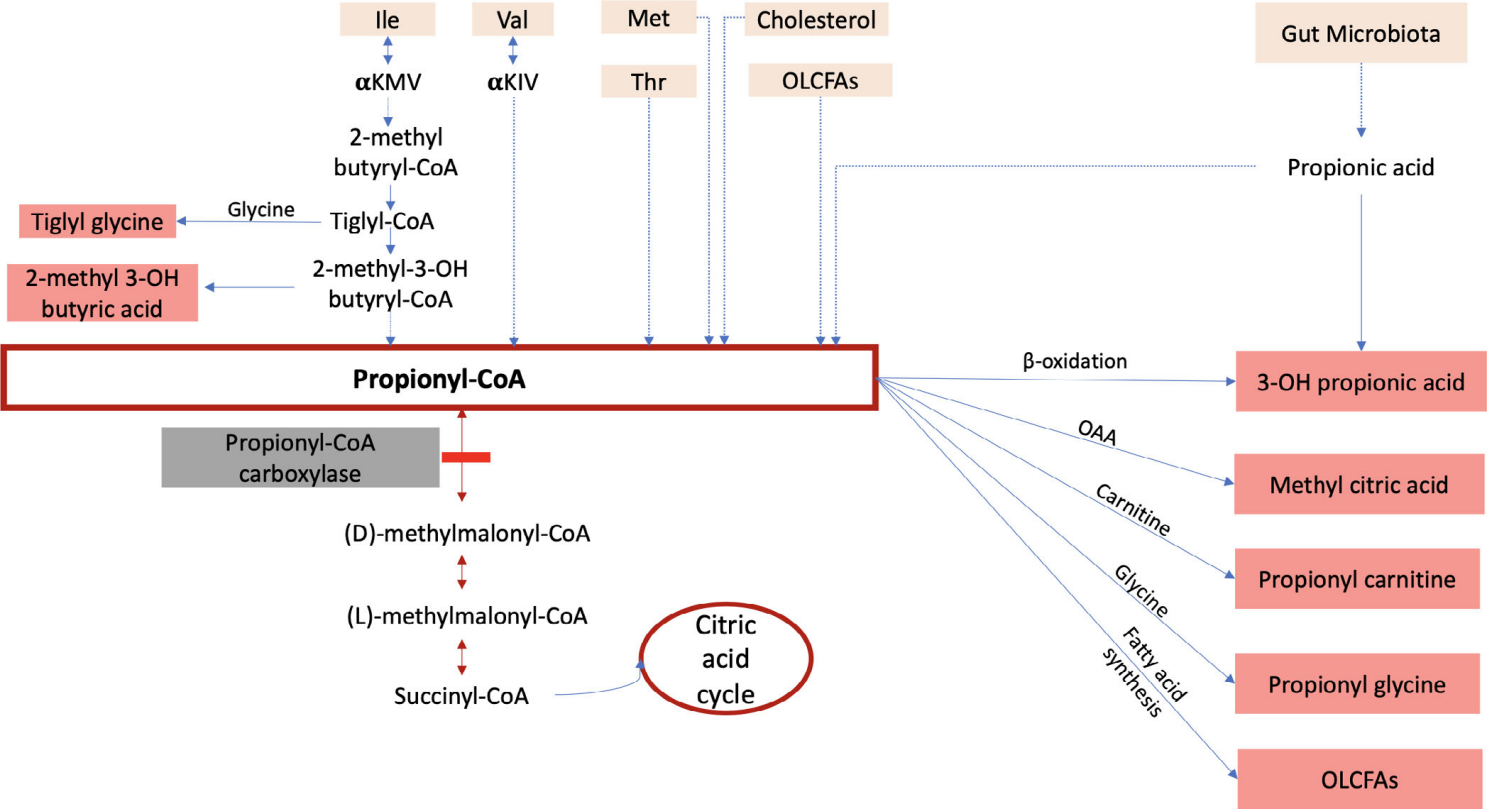
Metabolic pathways in propionic acidemia. Ile, isoleucine; Met, methionine; OAA, oxaloacetate; OLCFAs, odd‐numbered long‐chain fatty acids; Thr, threonine; Val, valine

PCC is composed by two distinct subunits (PCCA and PCCB) and mutations in either of their genes can produce PA. The etiopathology of PA is not completely understood, but apart from interference with the citric acid cycle, a large variety of secondary metabolites is formed that interfere in other metabolic pathways and are responsible for the severe metabolic acidosis, ketosis, hypoglycemia, lactic acidosis, and hyperammonemia observed in these patients. Many of these secondary metabolites are excreted in urine, such as 3‐hydroxypropionic acid or methyl citric acid (Figure 1).

Most of the patients with PA present in the first days of life with metabolic coma, lethargy, vomiting, dehydration, or seizures. Later in life, metabolic crisis can be triggered by infections, fever, or other catabolic events. The management of PA patients include low protein intake limiting the propiogenic amino acids (isoleucine, valine, methionine, and threonine), together with precursor‐free amino acid formulas in order to achieve WHO nutritional recommendations. Additionally, cofactors such as l‐carnitine and intermittent antibiotic therapy in order to avoid gut propionic formation are indicated.^1^, ^2^

Neonatal screening and a better control of decompensations have significantly increased the survival of PA patients over the last decades. However, chronic complications have become increasingly apparent even in patients with adequate metabolic control, posing new challenges in patients' care. These complications can affect any organ: epilepsy, movement disorders caused by metabolic stroke of the basal ganglia, optic neuropathy, cardiomyopathy, pancreatitis, high liver transaminase levels, anemia, and so on. The underlying mechanism of these complications is still debated.^3^

It is clear that PA patients, even those with an apparently good clinical status, require a close clinical and biochemical supervision. In order to improve the follow‐up of our patients and lower the burden of the families, we have designed a study in which we compared the traditional gold‐standard methods of liquid blood and urine analysis for the PA metabolites with the dried blood (DBS) and dried urine spots (DUS) that could be collected by the families in their homes. We also tried to identify which metabolites best correlate with the metabolic status of the patient.

## 2 MATERIALS AND METHODS

We designed a prospective study in which we analyzed data collected from PA patients followed in a single metabolic reference center in Spain from January 2015 to September 2017. The study was approved by the Ethical Committee of our hospital and all patients or their guardians signed the appropriate informed consent form. The study received financial support from an independent foundation (Fundación Ramon Areces).

Samples of DBS and DUS were collected monthly by the patient's family in their homes and sent to the laboratory by regular mail. Both dried spots and liquid blood and urine samples were collected every 3 months or during metabolic decompensation in the hospital. We defined metabolic decompensation as the presence of either hyperammonemia (ammonia levels > 60 μmol/L), ketosis (++), or metabolic acidosis (pH < 7.30, HCO_3_ < 20 mmol/L).

All lab measurements were completed in our ERNDIM approved, reference laboratory (CEDEM, Centro de Diagnostico de Enfermedades Moleculares, Universidad Autonoma, Madrid). Acylcarnitines and amino acids in DBS were determined as their butyl esters by flow injection analysis‐tandem mass spectrometry with stable isotope dilution.^4^, ^5^ Simultaneously, acylcarnitines were acquired by precursor ion scan of *m*/*z* 85 mode and amino acids by neutral loss of 102 amu, except basic amino acids, glycine, leucine, and isoleucine which were acquired by multiple reaction monitoring (MRM). Amino acids in plasma/serum were analyzed by ion‐exchange chromatography with ninhydrine. Organic acids and acylglycines in DUS were analyzed by HPLC‐tandem mass spectrometry and MRM mode.^4^ In addition, an ACE C18 HPLC column was included to resolve MRM isomers.^6^ Organic acids in liquid urine samples were determined as trimethylsilyl derivatives by GC‐MS after urease treatment and ethyl acetate liquid‐liquid extraction without oxymation. Odd long‐chain fatty acids in plasma/serum were analyzed by gas chromatography coupled to flame ionization detection.^7^ Total (free and esterified) fatty acids were obtained after acidic hydrolysis and derivatized as methyl esters. The results were given as the percentage of C15, C17, and C17:1 in the total fatty acid content.

The data collected was deidentified for statistical analysis. We evaluated the correlation coefficients between the liquid and the filter paper samples by linear regression and we included the decompensation as an interaction variable. We used multivariant model for the variables with statistically significant interaction and the univariant model for those with no significant interaction. The significance of the regression was established using STATA 15 and considered statistically significant for *P*‐value < .05.

## 3 RESULTS

All 10 PA patients attending the Metabolic Unit of the Hospital Ramon y Cajal (Madrid, Spain) agreed to participate in the study. Median age at the beginning of the study was 13.5 years (range: 3‐35 years). The clinical and demographic characteristics of our sample are shown in Table 1.

**TABLE 1:** Demographic and clinical data of 10 PA patients included in the study

	Sex	Current age (y)	Age at diagnosis	Genetics	Clinical course (long‐term complications)
1	M	Died (5)	Neonatal	PCCA gene p.Leu470Arg/p.Leu470Arg	Severe neuromotor delay Choreoathetosis, basal ganglia involvement, leukopenia, frequent infections, dilated cardiomyopathy, pancreatitis
2	M	9	Neonatal	PCCB gene p.Gly407Argfs*14/p.Arg410Trp	Severe neuromotor delay, anemia, leukopenia
3	F	35	Neonatal	PCCB p.Glu168Lys/p.? (c.183+3G>C)	Neuromotor delay
4	F	29	Neonatal	PCCB gene p.Gly407Argfs*14/p.Gly407Argfs*14	Neuromotor delay, dilated cardiomyopathy
5	F	15	6 mo	PCCB gene p.Arg512Cys/p.Gly255Ser	Neuromotor delay, epilepsy, pancreatitis, myositis
6	M	6	Neonatal screening	PCCB p.Asn536Asp/p.Asn536Asp	Autism
7	F	29	Neonatal	PCCB gene p.Gly407Argfs*14/p.Arg165Gln+p.Ala497Val	Peripheric neuropathy, neuromotor delay, pancreatitis, thrombopenia
8	F	24	4 mo	PCCB gene p.Gly407Argfs*14/p.Glu168Lys	—
9	F	12	4 mo	PCCA gene p.Gly477fs*9/p.Cys616_Val633del	Pancreatitis
10	M	10	6 mo	PCCA gene p.Gly477fs*9/p.Cys616_Val633del	Sever neuromotor delay

**FIGURE 2:**
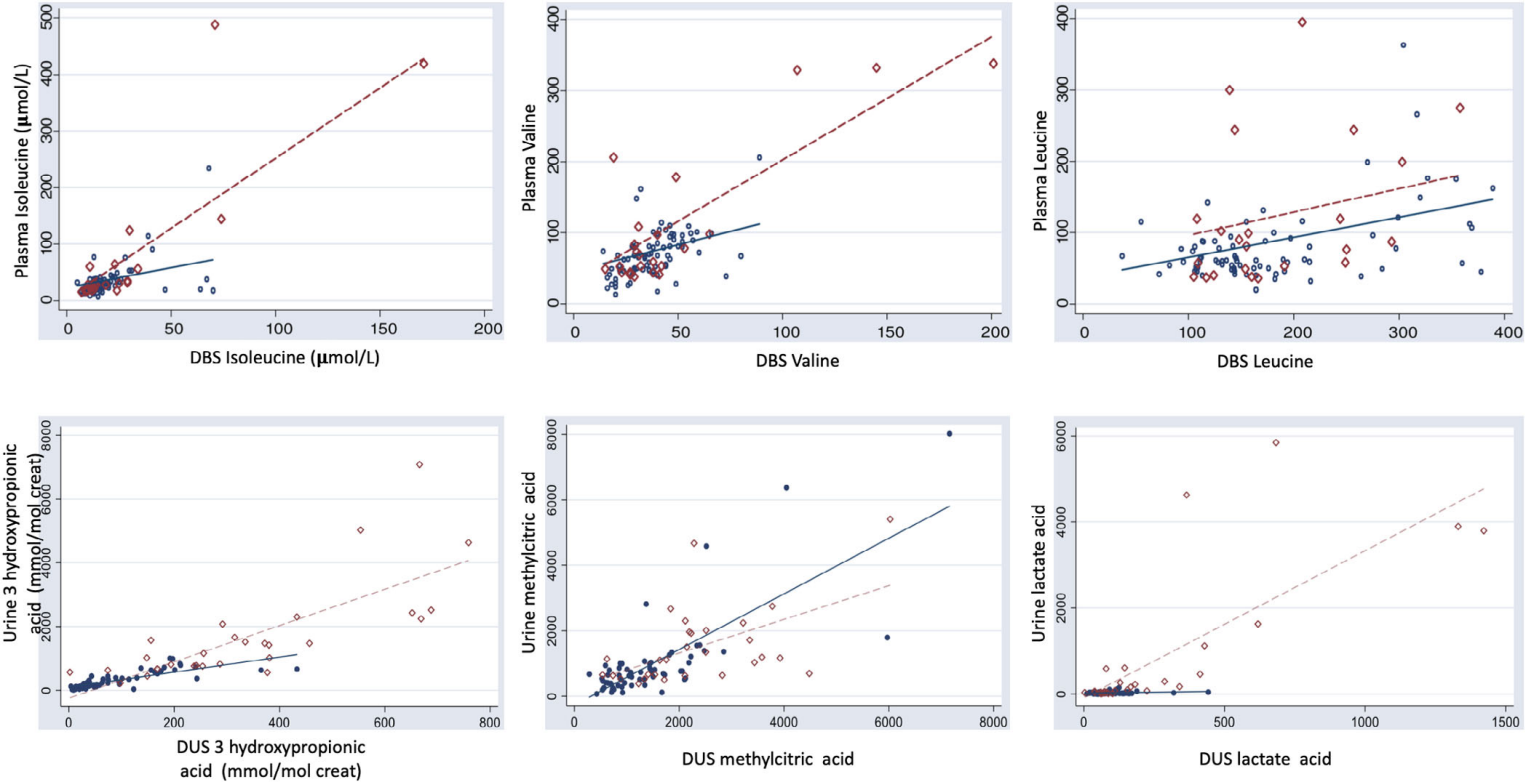
Association between the liquid (plasma/ urine) and the filter paper samples (DBS/DUS) using mixed linear regression models in metabolic stability (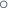) and crisis (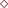). DBS, dried blood spot; DUS dried urine spot

**FIGURE 3:**
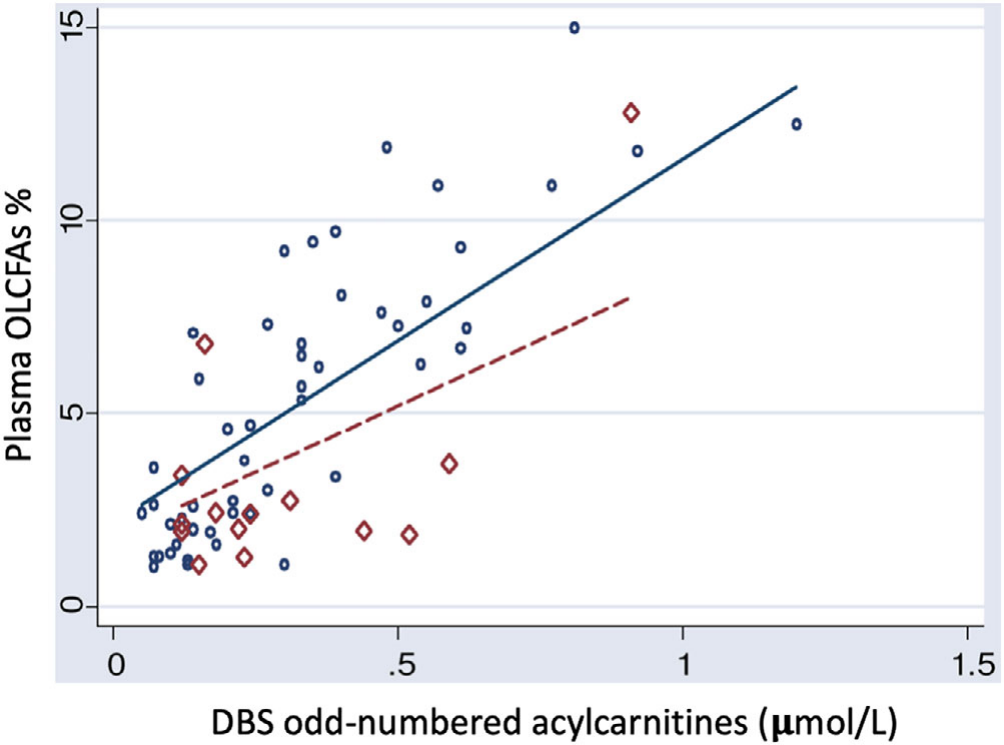
Association between plasma OLCFAs (%) and the DBS impar acylcarnitines sum using the mixed linear regression models in metabolic stability (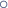) and crisis (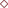). DBS, dried blood spot

We identified 40 metabolic crises during the study (18 with hyperammonemia, 22 with ketosis without hyperammonemia). The metabolic profile of our patients is shown in Figures 2 and 3 and summarized in Tables 1 and 2. Regarding the different metabolites measured, results are shown in Table 2. As a summary:

**TABLE 2:** Linear regression in multivariant model (significant interaction)

	Metabolic stability		Metabolic crisis	
	Regression coefficient	*P*‐value	Regression coefficient	*P*‐value
3‐OH propionic acid	2.36 (0.79;3.93)	.003	5.68 (4.63;6.73)	<.001
Methyl citric acid	0.85 (0.66;1.03)	.04	0.51 (0.24;0.77)	<.001
Citric/methyl citric ratio	0.86 (0.67;1,06)	<.001	−0.24 (−1.08;0.6)	.57
Lactate	0.08 (−1,88;2,06)	.93	3.41 (2.82;4.01)	<.001
2‐Methyl 3‐hydroxy butyric acid	0.08 (−0,7;0,87)	.83	1.08 (0.68;1.47)	<.001
Tiglyl glycine	1.94 (−0.9;4.8)	.18	6.97 (5.45;8.50)	<.001
Fumaric acid	4.8 (2.92; 6.68)	<.001	8.9 (7.06; 10.78)	<.001
Malic acid	0.80 (0.35;1.25)	<.001	2.00 (1.50; 2.45)	<.001
Isoleucine	0.7 (0.65;1.35)	.03	2.50 (2.00;3.00)	<.001
Leucine	0.30 (0.10;0.45)	<.001	0.30 (0,15; 0,65)	.04
Valine	0.75 (0.25;1.20)	.03	1.70 (1.45; 2.00)	<.001

*Amino acids*: branched‐chained amino acids (BCAA) in DBS were correctly estimated comparing to plasma (*P*‐value .03 for isoleucine and valine, *P*‐value .001 for leucine). During metabolic crisis there was a significant elevation of BCAAs (*P*‐value < .001 for isoleucine and valine, *P*‐value .04 for leucine), reflecting increased catabolism and ketogenesis. Other amino acids did not have such a good correlation in plasma and in DBS and no differences were observed during decompensations.

*Organic acids*: findings in DUS and in liquid urine correlated and reflect the metabolic status of the patients. As expected, there was an elevation of several urinary organic acids during the decompensation crisis, which was statistically significant in the case of 3‐hydroxypropionic acid (*P*‐value < .001), methyl citric acid (*P*‐value .04), lactate (*P*‐value < .001), tiglyl glycine (*P*‐value < .001), 2‐methyl 3‐hydroxy butyric acid (*P*‐value < .001), fumaric (*P*‐value < .001), and malic acid (*P*‐value < .001) (see Table 2). As for propionyl glycine, an important marker of PA, there was a good correlation in the univariant model between DUS and urine (*P*‐value < .001), since no significant interaction was detected.

*Acylcarnitines*: there was a good statistical correlation between free carnitine and propionyl carnitine levels in plasma and DBS samples in the univariant model (P‐value < .001 for both markers). No significant changes were found in any of the acylcarnitines during the metabolic crisis.

*Odd‐numbered long‐chain fatty acids*: OLCFAs have been used as a long‐term control marker in PA patients. It cannot be analyzed in DBS. However, odd‐numbered acylcarnitines (C14:1‐OH + C15, C16:1‐OH + C17, C16:2‐OH + C17:1) are expressed in DBS as a common peak. We observed a strong correlation between the sum of odd‐numbered acylcarnitines in DBS and the OLCFAs levels in plasma with a *P*‐value < 0.001 (CI 95% [5.41; 10.95], regression coefficient: 8.2) in the univariant model.

## 4 DISCUSSION

PA is one of the most frequent and severe types of organic acidemia and as such requires frequent clinical and biochemical controls that up to now could only be performed in a hospital setting. Adding to their regular visits, a majority of patients need to come to the hospital due to situations that have led or may lead to a metabolic decompensation. It is in the first years of life in which patients are especially at risk, as metabolic control is harder to achieve because of frequent infections or feeding difficulties. Whether because of previous frequent intravenous procedures or because of age, many times blood and urine collection are difficult to obtain in PA patients.

Also, the number of PA children who are surviving into adolescence and adulthood is increasing and more chronic complications are observed. These problems are usually not related with previous metabolic decompensations and their pathophysiology is not well understood. We cannot help but wonder if being able to have more frequent measurements of the metabolic status of our patients might help better understand the underlying mechanisms of long‐term complications and help achieve better outcomes, as it happened in diabetes and phenylketonuria. Long‐term management of PA is a difficult task, considering that no reliable biochemical parameters are yet available for quality control of therapy.^8^

Most of the PA literature is centered on the pathophysiology of the metabolic disturbances. However, there are also social and quality of life issues for the patients and their families which tend to be disregarded because our interventions are indispensable to avoid complications and frequently save the patient's life. One of the aims of our study was to determine whether the filter paper samples could be a method that reliably reflect the metabolic status of PA patients. If so, using this sample collection method would avoid many invasive procedures, might help increase the autonomy of the patients, who could send the paper samples from home. Easier sample handling and storage and the limitation of hospital visits are economically interesting. Therefore, the use of DBS and DUS would be more convenient for hospitals, laboratories, and patients.

In stable conditions, we observed a good correlation for BCAAs levels as well as for other specific PA metabolites such as 3‐hydroxypropionic acid, methyl citric acid, and propionyl carnitine. During the metabolic crisis, there was a significant elevation of BCAA, reflected both in DBS and plasma, as a marker of decompensation. Several metabolites are formed from the breakdown of isoleucine, such as tiglyl‐CoA which is trapped by glycine and excreted as tiglyl glycine.^9^ Hydration of tiglyl‐CoA results in formation of 2‐methyl 3‐hydroxy‐butyryl‐CoA, also significantly elevated during the decompensation. The amount of these former metabolites during the acute metabolic derangement therefore seems to reflect the enhanced metabolism of Isoleucine. Other tested parameters such as 3‐hydroxypropionic acid, methyl citric acid, lactate, and propionyl carnitine, also showed a significant correlation during the acute decompensation.

Although there is little consensus concerning the utility of metabolic markers in the long‐term follow‐up, quantitative plasma amino acids are indicated each 3 to 6 months mainly to evaluate the nutritional status.^1^ Low levels of Isoleucine are often described in PA forms that require severe natural protein restriction and are associated with acrodermatitis enteropathica‐like, requiring supplementation.^1^ In our series, 9 of 10 receive isoleucine supplements to maintain Ile levels above 15 μmol/L in order to avoid the skin lesions. Results from DBS samples correctly reflected the nutritional status, with a strong correlation both in stable condition as in metabolic acute decompensation.

Other amino acids patterns, such as high glycine or lysine, low levels of citrulline or normal/low glutamine during the hyperammonemia,^10^ that might help in understanding the biochemical changes in PA, have not proved any clinical relevance.

Free carnitine C0 and propionyl carnitine C3 were strongly correlated in the univariant model in plasma and DBS, but no interaction with the decompensation was observed. All our patients were supplemented with l‐carnitine (150‐200 mg/kg/d), as all of them were diagnosed previously to be included in the study.

One important limitation of our study was the lack of measure of the ketone bodies in the DUS due to the extreme volatility of acetoacetate and 3OH butyric acid. However, the ketones bodies can be easily determined qualitatively in dipstick urine.

Utilization of propionyl‐CoA as a primer in fatty acid synthesis instead of acetyl‐CoA leads to accumulation of OLCFAs in patients with PA, such as C15 and C17 fatty acids. These acids, like any other carboxylic acid, could be esterified to form carnitine‐conjugates, among them the odd‐number acylcarnitines detected in DBS samples of our patients. It has been proposed that the content of OLCFAs in different body lipids or tissues (ex. erythrocyte membrane) can be used as a tool for monitoring patients with PA, as a quantitative estimation of the intracellular pool of propionyl‐CoA.^11^, ^12^ The sum of the OLCFAs 15‐ and 17‐carbon saturated and 17‐carbon monounsaturated fatty acids (C15:0, C17:0, C17:1) is calculated and expressed as a percentage of the total C14‐C22 fatty acids. The technique was first described in erythrocyte, but it can also be performed in plasma.

It is known that OLCFAs production is already active prenatally and large amounts have been detected in fetuses suffering of propionate metabolism defects, even if these alterations seem not to influence normal pregnancy and birth.^13^ Enhanced postnatal lipolysis may lead to increase production of propionyl‐CoA in mitochondria not only from proteins but also from OLCFAs.^9^ Thus, not surprisingly, several papers have recently reported that in patients affected by disorders of propionate metabolism, newborn screening showed an increased concentration of heptadecanoyl carnitine (C17) in addition to elevated C3; therefore, C17 has been proposed as a second‐tier test in the neonatal screening for the detection of propionate metabolism defects.^14^

In our study we detected in DBS samples elevation of odd number acylcarnitines that were expressed as a common peak C14:1‐OH + C15, C16:1‐OH + C17, C16:2‐OH + C17:1. We could also demonstrate a strong statistical correlation between the sum of odd‐numbered acylcarnitines and the OLCFAs in plasma. As the OLCFAs have shown utility as a biochemical marker for the severe forms of PA, we could conclude that the sum of odd‐numbered acylcarnitines might be considered an equivalent of OLCFAs in DBS. The usefulness of these parameters in monitoring PA patients or as a second‐tier test in neonatal screening of propionate disorders should be further analyzed.

Despite many advances that have been made in understanding the disorders of propionate metabolism, still no clinical or biochemical marker is available for the quality of treatment control. A recent retrospective study concluded that in decision‐making on the start of emergency treatment in PA patients, the most useful biochemical parameters seem to be ammonia, acid‐base balance and anion gap; no statistical correlation was found with any other biochemical markers of PA. Still, this study refers exclusively to the risk of metabolic decompensation, the question of the long‐term management and outcome remain unresolved.^15^

## 5 CONCLUSIONS

PA patients require lifelong monitoring. DBS and DUS correctly reflect the metabolic and nutritional status and might be used for the long‐term follow‐up of these patients. The simplicity of obtention and transportation of the samples are considerable advantages. Their use can facilitate having more frequent metabolic control of patients and are especially convenient in those cases in which the patient resides far from the metabolic center or have a difficult social situation.

## CONFLICT OF INTEREST

Sinziana Stanescu has received travel and speaker fees from Nutricia, Mead Johnson, Genzyme, Recordatti Rare Diseases, Vitaflo‐Nestlé, BioMarin. Amaya Belanger has received travel and speaker fees from Nutricia, Mead Johnson, Genzyme, Recordatti Rare Diseases, Vitaflo‐Nestlé, Takeda, BioMarin; advisory fees from BioMarin and Merk Serono. Francisco Arrieta has received travel and speaker fees from Nutricia, Mead Johnson, Recordatti Rare Diseases, Vitaflo‐Nestlé, BioMarin. Mercedes Martínez‐Pardo has received travel and speaker fees from Nutricia, Mead Johnson, Genzyme, Recordatti Rare Diseases, Vitaflo‐Nestlé, Takeda, BioMarin. All other authors declare no conflicts of interest.

## AUTHOR CONTRIBUTIONS

Dr Martinez‐Pardo had a significant contribution to the conception and design of the study. Drs Stanescu and Belanger‐Quintana had contributed to the acquisition and interpretation of the data. Dr Fernandez‐Felix had performed the statistical analysis. All authors have contributed to the acquisition and the interpretation of the data and gave the final approval for the article.

## ETHICS STATEMENT

This article does not contain any studies with human or animal subjects performed by any of the authors. The study was approved by the Ethical Committee of our hospital (Hospital Universitario Ramon y Cajal, Madrid, Spain). The patients signed a consent statement previous to the inclusion in the study.
